# Administration of N-Acetylcysteine to Regress the Fibrogenic and Proinflammatory Effects of Oxidative Stress in Hypertrophic Ligamentum Flavum Cells

**DOI:** 10.1155/2022/1380353

**Published:** 2022-10-26

**Authors:** Yu-Chia Hsu, Hao-Chun Chuang, Kun-Ling Tsai, Ting-Yuan Tu, Yan-Jye Shyong, Cheng-Hsiang Kuo, Yuan-Fu Liu, Shu-Shien Shih, Cheng-Li Lin

**Affiliations:** ^1^Department of Orthopaedic Surgery, National Cheng Kung University Hospital, College of Medicine, National Cheng Kung University, Tainan, Taiwan; ^2^Department of Physical Therapy, College of Medicine, National Cheng Kung University, Tainan, Taiwan; ^3^Department of Biomedical Engineering, National Cheng Kung University, Tainan, Taiwan; ^4^Department of Clinical Pharmacy and Pharmaceutical Sciences, National Cheng Kung University, Tainan 70101, Taiwan; ^5^Department of Biochemistry and Molecular Biology, National Cheng Kung University, Tainan, Taiwan

## Abstract

Ligamentum flavum hypertrophy (LFH) is a major cause of lumbar spinal stenosis (LSS). In hypertrophic ligamentum flavum (LF) cells, oxidative stress activates intracellular signaling and induces the expression of inflammatory and fibrotic markers. This study explored whether healthy and hypertrophic LF cells respond differently to oxidative stress, via examining the levels of phosphorylated p38 (p-p38), inducible nitric oxide synthase (iNOS), and *α*-smooth muscle actin (*α*-SMA). Furthermore, the efficacy of N-acetylcysteine (NAC), an antioxidant, in reversing the fibrogenic and proinflammatory effects of oxidative stress in hypertrophic LF cells was investigated by assessing the expression levels of p-p38, p-p65, iNOS, TGF-*β*, *α*-SMA, vimentin, and collagen I under H_2_O_2_ treatment with or without NAC. Under oxidative stress, p-p38 increased significantly in both hypertrophic and healthy LF cells, and iNOS was elevated in only the hypertrophic LF cells. This revealed that oxidative stress negatively affected both hypertrophic and healthy LF cells, with the hypertrophic LF cells exhibiting more active inflammation than did the healthy cells. After H_2_O_2_ treatment, p-p38, p-p65, iNOS, TGF-*β*, vimentin, and collagen I increased significantly, and NAC administration reversed the effects of oxidative stress. These results can form the basis of a novel therapeutic treatment for LFH using antioxidants.

## 1. Introduction

Lumbar spinal stenosis (LSS) was first described in 1900 [1], and its symptoms include backache, numbness, or bladder disturbance [2]. It is a common disease with an estimated prevalence of 19.4% in people in their sixties [3]. Ligamentum flavum hypertrophy (LFH) is considered a major cause of LSS [4]. LFH is usually treated with oral analgesics, surgical intervention, and rehabilitation [5]. However, oral analgesics such as nonsteroidal anti-inflammatory drugs have multiple adverse effects, including gastrointestinal bleeding and kidney function impairment. Therefore, developing novel pharmacological therapies for LFH with relatively minimal side effects is imperative. Recent studies have revealed multiple pathomechanisms for LFH, including histologic changes that involve elevated collagen and diminished elastic fiber content, increased inflammatory cytokine levels (e.g., inducible nitric oxide synthase (iNOS), matrix metalloproteinase (MMP), interleukin- (IL-) 6, and IL-8 levels), increased growth factor expression (e.g., transforming growth factor- (TGF-) *β* and vascular endothelial growth factor expression), and focal angiogenesis [6–13]. However, despite the known etiologies, a targeted therapy for LFH has yet to be developed.

Recently, elevated oxidative stress and increased oxidative DNA damage have been observed in patients with LFH [14–16]. In 2020, one study noted increased production of reactive oxygen species (ROS) and decreased levels of endogenous antioxidants such as glutathione (GSH) and superoxide dismutase in hypertrophic ligamentum flavum (LF) cells; the study also revealed that under oxidative stress stimulation, the upregulation of intracellular signaling pathways and elevated expression levels of fibrotic and inflammatory markers were identified [17]. Oxidative stress is a major pathogenic factor in the development of LFH as well as in other diseases such as Parkinson disease (PD), acute lung injury, ischemic heart disease, chronic kidney disease, liver cirrhosis, and pelvic organ prolapse [18–26].

Considering the role of oxidative stress in disease development, studies have administered antioxidants such as N-acetylcysteine (NAC), curcumin, *β*-carotene, vitamins, coenzyme Q10, and folic acid for therapy and have noted diverse clinical responses depending on the disease. Moreover, antioxidants have been applied for treating diseases associated with a wide range of organs or systems, including the brain, lungs, heart, vascular system, liver, and kidneys [18, 20, 23, 27–30]. Accordingly, we conducted this study with the aim of exploring the effects of an antioxidant, namely, NAC, on LFH. Our study is the first to apply an antioxidant agent for LFH therapy. We chose NAC because of its ability to increase GSH precursors [31] and its minimal adverse effects, especially when compared with oral analgesics [32].

Because of the association between oxidative stress and LFH, NAC is proposed as a possible treatment option for LFH. In this study, we first compared the response of healthy LF cells and that of pathologically hypertrophic LF cells to oxidative stress. The inflammatory and intracellular signaling in hypertrophic LF cells was hypothesized to respond more significantly compared with their healthy counterparts. Secondly, we evaluated the ameliorative effects of NAC on the blockage of oxidative stress-induced inflammation and fibrosis in hypertrophic LF cells.

## 2. Methods

### 2.1. Participants and Magnetic Resonance Imaging Analysis

**Figure 1:**
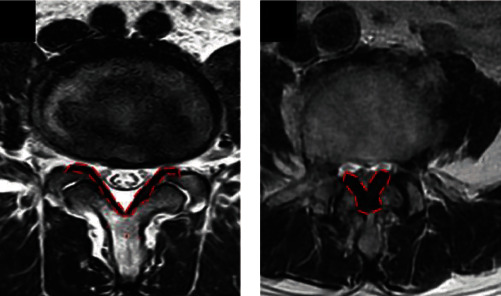
MRI scans of the lumbar spine. T2-weighted MRI scans of the LF (surrounded by orange broken lines) of (a) a patient with LDH and (b) a patient with LSS.

After excluding patients with a history of epidural or selective nerve-root blocks, malignancy, vertebral fracture, vertebral osteomyelitis, or previous spine surgery, we included 52 patients undergoing spinal surgery at a tertiary referral hospital in southern Taiwan. Patient characteristics for LFH were obtained from other studies [33, 34]. All patients underwent magnetic resonance imaging (MRI) preoperatively. The maximum thickness of the LF was measured using axial T2-weighted images acquired at the facet-joint level of the lesion (Figure 1) [35]. The radiographic analyses were performed by two senior spine surgeons independently, who were not involved in providing clinical care to the patients. The surgeons each measured the thickness of the LF twice, and the average of the four measurements was used as the final result.

We aseptically collected hypertrophic LF specimens from 28 patients with LSS (LSS group) during posterior lumbar decompression surgery. Moreover, we collected healthy LF tissue samples from 24 patients with lumbar disc herniation (LDH; LDH group) during lumbar discectomy surgery. The LF at the diseased lumbar level was yielded en bloc, and the epidural fat and bone-ligament junction were accurately removed. Fifteen specimens from the LDH group and 40 specimens from the LSS group were used for LF cell isolation.

The study was conducted according to the Declaration of Helsinki and approved by the Institutional Review Board of National Cheng Kung University Hospital (A-ER-108-541, A-ER-110-001). The protocol in the study was reviewed by the Protection of Human Subjects Committee. All 52 patients recruited provided written informed consent.

### 2.2. Human LF Cell Characterization

**Figure 2:**
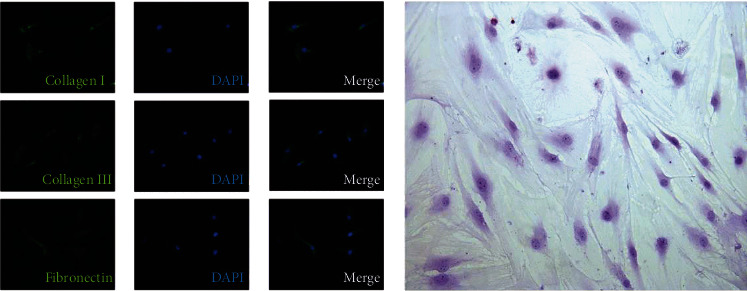
(a) Immunofluorescence analysis of ligamentum flavum cells. Immunofluorescence was visualized using FITC (green) and the nuclei by DAPI (blue). Cells cultured in the current study expressed collagen type I, collagen type III, and fibronectin. (b) The alkaline phosphatase activity was assessed by BCIP-NBT (Sigma). The alkaline phosphatase activity was detected intracellularly in over 80% of cells, the morphology of which was primarily spindle-shaped and polygonal.

In this study, human ligamentum flavum cells were characterized by high levels of alkaline phosphatase activity, production of a matrix rich in type I and III collagen, expression of fibronectin, and a spindle or polygonal morphology, as described by Specchia et al. [36, 37]. We performed immunofluorescence analyses of collagen type I, collagen type III, and fibronectin, which were visualized using FITC (green) and the nuclei by DAPI (blue). Also, the alkaline phosphatase activity was assessed by exposing fixed cultures for 30 minutes to a solution containing BCIP-NBT (Sigma). Cells cultured in the current study expressed collagen type I, collagen type III, and fibronectin and were positive for alkaline phosphatase activity. The morphology of ligamentum flavum cells was primarily spindle-shaped and polygonal as previous studies (Figure 2).

### 2.3. Human LF Cell Isolation and H_2_O_2_ and NAC Administration

First, the LF specimens were washed with phosphate-buffered saline until all the residual adipose or connective tissues had been fully removed. The specimens were then minced into 0.5 mm^3^ pieces and placed in a 10 cm culture dish with 10 mL of high-glucose Dulbecco's modified Eagle's medium (Gibco, Melbourne, Australia) with 10% fetal bovine serum (Gibco) and 100 U/mL of penicillin. The specimens were incubated at 37°C in an air-humidified incubator containing 5% CO_2_, and the culture medium was changed twice weekly. When the LF cells reached an appropriate confluence in the dishes, they were treated with 0.25% trypsin and subcultured in accordance with the aforementioned protocols. After the third passage, the derived cells were used for the experiments. The cells derived from the LSS patients were seen as hypertrophied LF cells, while the ones derived from the LDH patients were regarded as healthy ones. The former was the experimental group, and the latter was the control group. The descriptions came from the previous studies for LFH [4, 37]. Before proceeding to experiments, cell characterization was performed. In the experiments, 25 *μ*M H_2_O_2_ was selected to induce oxidative stress, as described in other studies [38, 39]. Furthermore, 10 mM NAC was administered as the oxidative scavenger and antioxidant precursor for ROS suppression; the NAC concentration was also based on the suggestions of other cell-related studies [38, 40–43].

### 2.4. Quantification of ROS Activity in Hypertrophic LF Cells

The ROS levels were measured using 2′,7′-dichlorodihydrofluorescein diacetate (DCFH-DA), a fluorescent dye that can detect H_2_O_2_, hydroxyl and peroxyl radicals, peroxynitrite anions, and other ROS activities in cells. The LF cells isolated from the LSS specimens were placed in a 6 cm dish and divided into three groups: the first group comprised cells without treatment (control group), the second group comprised cells subjected to 30 min or 24 h of 25 *μ*m H_2_O_2_ treatment (H_2_O_2_ treatment group), and the third group comprised cells subjected to 30 min of pretreatment with 10 mM NAC and then stimulation with H_2_O_2_ (NAC pretreatment–H_2_O_2_ stimulation group). All cells were stained with 25 *μ*m of DCFH-DA (ab113851, DCFDA cellular ROS detection assay kit) for 30 min at 37°C, and the ROS levels were measured after stimulation with H_2_O_2_, with or without NAC suppression, by using a florescence microplate reader (Ex/Em = 485/535 nm). Laboratory data were analyzed using an unpaired *t*-test.

### 2.5. Western Blot Analysis

**Figure 3:**
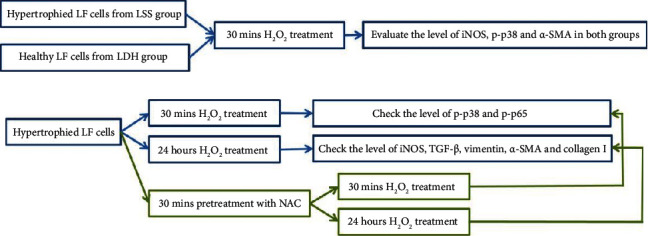
The graphical scheme of the study. (a) The third passage of cultured LF cells after cell characterization was used in the study. The hypertrophied LF cells from LSS group and healthy ones from LDH group were exposed to H_2_O_2_, and the level of inflammatory factors (iNOS), fibrotic marker (*α*-SMA), and intracellular signaling (p-p38) before and after oxidative insult would be evaluated. (b) In hypertrophied LF cells, after 30 minutes or 24 hours of H_2_O_2_ treatment, the response to oxidative stress would be checked via quantifying the level of p-p38, p-p65, iNOS, TGF-*β*, vimentin, *α*-SMA, and collagen I as illustrated in the blue square and arrow. For the evaluation of the effect of antioxidant (NAC) to regress the response to oxidative stress, 30 minutes pretreatment with NAC would be finished before exposure to H_2_O_2_, and the expression of protein was quantified after NAC and H_2_O_2_ as illustrated in the green square and arrow.

After treatment with H_2_O_2_ with or without NAC, the tissue was lysed in ice-cold lysis buffer (1 : 10; *w*/*v*) containing 20 mM 4-(2-hydroxyethyl)-1-piperazineethanesulfonic acid (pH 7.2), 10% glycerol, 1% Triton X-100, 10 *μ*g/mL of leupeptin, 10 *μ*g/mL of aprotinin, and 1 mM phenylmethylsulfonyl fluoride. The solution was centrifuged at 12,000 rpm for 10 minutes, and the protein concentration was measured using a protein assay dye (Bio-Rad Laboratories, Hercules, CA, USA), with bovine serum albumin as the standard. We added 8% and 10% sodium dodecyl sulfate polyacrylamide gel for electrophoresis and then transferred the samples to nitrocellulose sheets (NEN Life Science Products, Boston, MA, USA) in a transfer apparatus (Bio-Rad) running at 1.2 A for 2 h. After blocking the blots with 5% nonfat skimmed milk in Tween 20, we added primary antibodies against the target proteins (GPX-1, TGF-*β*, iNOS, *β*-actin, p38, p-p38, p65, p-p65, *α*-SMA, collagen I, and vimentin) and then again added anti-rabbit IgG conjugated with alkaline phosphatase (dilution 1 : 5000; Jackson Immuno Research Laboratories, Philadelphia, PA, USA). Immunoblots were developed using 5-Bromo-4-chloro-3-indolyl phosphate/nitro blue tetrazolium solution (Kirkegaard and Perry Laboratories, Baltimore, MD, USA). The proteins were quantified through densitometry using the ImageJ computer program (National Institutes of Health; available at http://rsb.info.nih.gov/ij/). The following antibodies were used in this study: collagen I (Abcam, ab88147), iNOS (Abcam, ab178945), p38 (GeneTex, GTX110720), phosphorylated p38 (p-p38; Abcam, ab195049), p-p65 (Cell Signaling Technology, 3033), p65 (Cell Signaling Technology, 6956), *α*-smooth muscle actin (SMA; Abcam, ab7817), anti-*β*-actin (Thermo Fisher Scientific), anti-GPx-1/2 (SANTA CRUZ, sc-133160), anti-TGF-*β* (Santa Cruz Biotechnology, sc-130348), and anti-vimentin (Santa Cruz Biotechnology, sc-6260). The graphical scheme of the study is shown in Figure 3.

### 2.6. Statistical Analysis

Data on the patients' characteristics are presented as the mean ± standard deviation. An independent *t*-test and a chi-square test were used to compare the LDH and LSS groups. A one-way analysis of variance (ANOVA), followed by Tukey's post hoc test, was used to analyze data regarding cell cultures harvested at different time points. All data in this study were analyzed using SPSS (version 17; SPSS, Chicago, IL, USA).

## 3. Results

### 3.1. LSS Patients Were Older and Had More Hypertrophic LF Tissues Than Did LDH Patients

We included 52 patients in this study, of whom 28 were in the LSS group (undergoing decompression surgery for lumbar spinal stenosis) and the remaining were in the lumbar disc herniation (LDH) group. The mean ages of the patients in the LSS and LDH groups were 67.8 ± 9.2 and 43.1 ± 17.8 years, respectively (Table 1); therefore, the patients in the LSS group were significantly older than those in the LDH group (*p* < 0.001). In addition, the mean LF thickness in the LSS group was significantly higher than that in the LDH group (5.1 ± 0.7 vs.2.7 ± 0.4 mm; *p* < 0.001). In terms of sex, the LSS group exhibited a significantly higher female predominance than did the LDH group. The two groups did not differ significantly in terms of body mass index (*p* = 0.707), diabetes mellitus (*p* = 0.19), hypertension (*p* = 0.177), respiratory disease (*p* = 0.911), or smoking incidence (*p* = 0.175). LF thickness was weakly associated with age and sex, consistent with the report of a previous study [44]. In summary, the patient population included in this study was considered to be a representative sample because of the compatibility of its characteristics with those reported by previous epidemiologic studies [3, 45].

### 3.2. Intracellular Signaling and Inflammation Were More Active in Hypertrophic LF Cells

**Figure 4:**
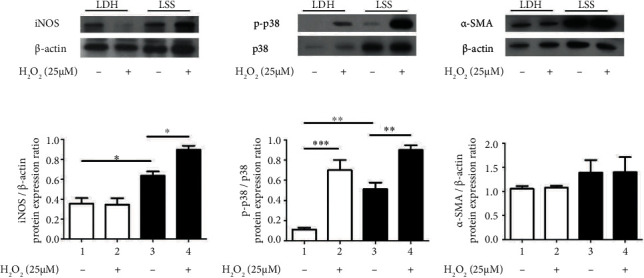
Relative protein expression levels indicating more active intracellular signaling and inflammatory responses in hypertrophic LF cells. (a) Protein expression of the p-p38/MAPK pathway and inflammatory and fibrotic markers determined through Western blotting. (b) Under oxidative stress, the protein expression ratio of iNOS increased only in the hypertrophic LF cells. (c) The protein expression ratio of p-p38 exhibited a dramatic increase in both the hypertrophic and healthy LF cells after H_2_O_2_-induced oxidative stress. (d) The expression of *α*-SMA did not increase after 30 min of H_2_O_2_ treatment in either group (*n* = 5; ^∗^*p* < 0.05 compared with the LDH control group or LSS control group; ^∗∗^*p* < 0.01 compared with the LDH control group or LSS control group; ^∗∗∗^*p* < 0.001 compared with the LDH control group; values were derived from a one-way ANOVA and Tukey's post hoc test).

After the cells were subcultured until the third passage, hypertrophic and healthy LF cells were exposed to H_2_O_2_ treatment for 30 min to stimulate oxidative stress. Before oxidative stress stimulation, the background levels of p38 MAPK signaling and iNOS were significantly higher in the hypertrophic LF cells than in the healthy cells, indicating that the hypertrophic LF cells had proinflammatory properties and more active intracellular signaling. After H_2_O_2_ treatment, the p-p38 and iNOS levels in the hypertrophic LF cells exhibited a more evident increase than did those in the healthy LF cells. In the healthy LF cells, the p-p38 levels increased significantly but the iNOS levels did not. Notably, the fibrotic response (*α*-SMA) did not increase after 30 min of H_2_O_2_ treatment in either group (Figure 4).

### 3.3. Increased Oxidative Stress Capacities and Decreased Antioxidant Markers Were Present in Hypertrophic LF Cells after H_2_O_2_ Treatment, Which Was Reversed through Antioxidant (NAC) Administration

**Figure 5:**
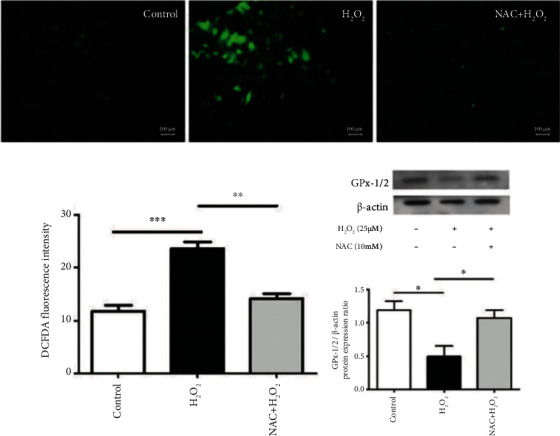
ROS activities in hypertrophic LF cells were induced by H_2_O_2_ stimulation and partially blocked by NAC. (a) ROS in cells were stained with DCFH-DA and measured using a microplate reader. The green fluorescence intensity indicated ROS generation. Compared with the control group, the H_2_O_2_ treatment group exhibited enhanced ROS expression, and this phenomenon was suppressed by NAC. (b) ROS levels in the LF cells were quantified, and the H_2_O_2_ treatment group had a significant increase in ROS levels compared with the control group. The NAC pretreatment-H_2_O_2_ stimulation group exhibited significantly lower ROS levels compared with the H_2_O_2_ treatment group. (c) GPX-1/2 expression levels in the cells were also assessed. The antioxidant capacity was significantly inhibited through H_2_O_2_ treatment and elevated through NAC pretreatment (*n* = 4; ^∗^*p* < 0.05 compared with the control group or H_2_O_2_ treatment group; ^∗∗^*p* < 0.01 compared with the H_2_O_2_ treatment group; ^∗∗∗^*p* < 0.001 compared with the control group; values were derived from a one-way ANOVA and Tukey's post hoc test).

Under H_2_O_2_-induced oxidative stress, the levels of ROS production were significantly elevated and the expression of GPX-1/2, a key intracellular antioxidant, was significantly inhibited in the H_2_O_2_ treatment group compared with the control group. In the NAC pretreatment-H_2_O_2_ stimulation group, ROS production decreased and GPX-1/2 levels increased. This indicates that NAC functions as an oxidative scavenger and antioxidant replenisher in hypertrophic LF cells, countering H_2_O_2_-induced oxidative stress (Figure 5). This finding is consistent with that of another study [46].

### 3.4. Administration of NAC Reversed the Proinflammatory and Fibrogenic Effects of Oxidative Stress in Hypertrophic LF Cells

**Figure 6:**
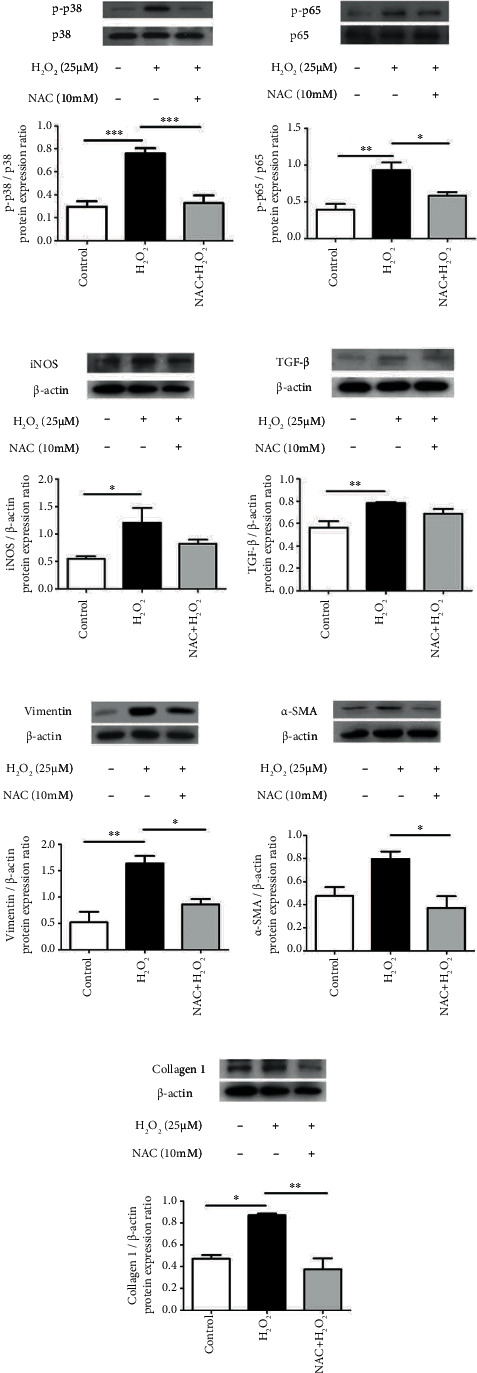
Protein expression of inflammatory cytokines, intracellular signaling, and fibrotic markers under H_2_O_2_ treatment with or without NAC. Under 25 mM H_2_O_2_-induced oxidative stress for 30 min, significantly elevated protein expression levels of p-p38 and p-p65 were detected, and under 25 mM H_2_O_2_-induced oxidative stress for 24 h, significantly elevated protein expression levels of iNOS, TGF-*β*, vimentin, and collagen I were also detected. After a 30 min pretreatment with 10 mM NAC, these markers were all significantly suppressed, except for iNOS and TGF-*β*. In spite of statistical non-significance, iNOS and TGF-*β* also showed a trend of diminish with NAC administration. (*n* = 5; ^∗^*p* < 0.05 compared with the control group or H_2_O_2_ stimulated group; ^∗∗^*p* < 0.01 compared with the control group or H_2_O_2_ stimulated group; ^∗∗∗^*p* < 0.001 compared with the control group or H_2_O_2_ stimulated group; values were derived from a one-way ANOVA and Tukey's post hoc test).

Under H_2_O_2_-induced oxidative stress, the hypertrophic LF cells exhibited significantly elevated levels of inflammatory cytokines (including iNOS and TGF-*β*), expression of activated intracellular signaling pathways (including p-p38 and p-p65), and levels of fibrotic markers (including collagen I and vimentin). The expression level of *α*-SMA also showed a trend of elevation with H_2_O_2_ treatment. NAC administration led to a decrease in oxidative stress-induced inflammation, intracellular signaling, and fibrosis (Figure 6). These results are consistent with those of another study that reported a positive association between LFH and oxidative stress-induced inflammation, intracellular signaling, and fibrosis [17]. Additionally, the results suggest that the proinflammatory and fibrogenic effects of oxidative stress in hypertrophic LF cells could be partially reversed by antioxidants.

## 4. Discussion

**Figure 7:**
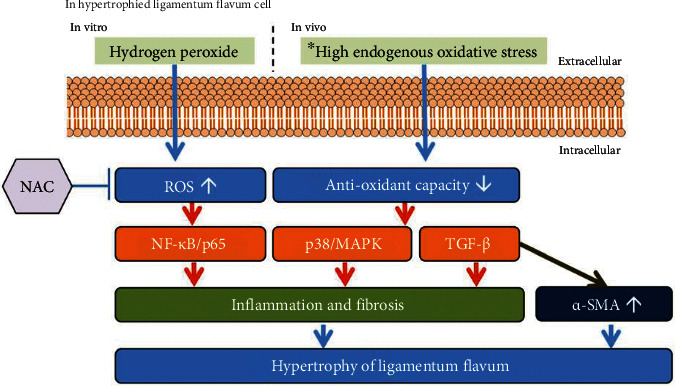
Oxidative stress-related molecular mechanism of LFH, which is blocked by NAC, an oxidant scavenger and antioxidant replenisher. In hypertrophic LF cells, under H_2_O_2_-induced oxidative stress, elevated ROS, and diminished antioxidant levels were detected. With oxidative stress, the expression levels of intracellular signaling pathways and growth factors were enhanced, inducing an increase in inflammatory and fibrotic markers. Inflammation, fibrosis, and *α*-SMA expression contribute to LFH formation. ^∗^Levels higher than baseline endogenous oxidative stress levels in hypertrophic LF cells compared with healthy cells derived from another study [17].

This study revealed that intracellular signaling activities and inflammatory markers in hypertrophic LF cells were consistently higher than those in healthy LF cells. These hypertrophic LF cells exhibited a more marked response against oxidative stress than did the healthy cells. Under H_2_O_2_-induced oxidative stress, the increase in ROS production along with the decrease in reducing substances induced the activation of intracellular signaling and the expression of inflammatory and fibrotic markers; these findings are consistent with the results of another study [17]. NAC administration effectively suppressed oxidative stress markers, replenished antioxidant capacity, and curbed the expression of proinflammatory and fibrogenic mediators. Moreover, NAC promoted detoxification, which may be related to the stimulation of GSH biosynthesis. Overall, oxidative stress engendered an increase in the expression of intracellular signaling pathways and inflammatory and fibrotic markers associated with the development of LFH, and this increase was countered by the administration of the antioxidant agent, NAC (Figure 7). These results indicate that NAC has potential as a novel therapy for LFH.

Oxidative stress imbalance is considered a factor in the development of several diseases, including Alzheimer's disease, PD, fibrosing alveolitis, acute lung injury, liver disease, and vascular disease [47–49]. Therefore, studies have explored the potential clinical application of antioxidants such as NAC; vitamins A, C, and E; folic acid; curcumin; and pyrolidium dithiocarbamate [18, 20, 28, 29, 50, 51]. Regarding PD, studies have reported that decreased nigral GSH levels were strongly associated with disease progression and that intravenous and oral NAC administration engendered a significant increase in serum GSH levels and significant improvements in clinical symptoms [20, 52]. Regarding fibrosing alveolitis, a study noted inappropriate oxidative stress and reduced antioxidant marker expression due to overactive phagocytes; nevertheless, oral NAC administration led to the recovery of antioxidant markers and improvements in pulmonary function [28]. Regarding acute lung injury, intravenous NAC administration led to improvements in oxygenation through the replenishment of reducing substances [24]. A study demonstrated higher ROS levels and lower antioxidant capacity levels (GSH) in hypertrophic LF cells; the study also noted that oxidative stress was correlated with inflammation and fibrosis [53]. Other studies have confirmed that NAC could replenish GSH biosynthesis without the blockage of negative feedback from buthionine sulfoximine [46, 54, 55]. Our study revealed similar results. Specifically, we observed that NAC partially blocked H_2_O_2_-induced ROS production and replenished depleted GPX-1/2, a reducing substance. In addition, NAC inhibited oxidative stress-induced inflammation, intracellular signaling, and fibrosis. These results indicate that NAC can protect LF cells against acute oxidative insult. Our study is the first to explore the use of an antioxidant as a treatment for LFH. The study expands the applications of NAC in oxidative stress-related diseases, including LFH. Long-term intake of NAC might ameliorate the depletion of reducing substances in hypertrophic LF cells, such as in PD [20]. However, the potential preventive effects of NAC against LFH should be further explored. Since 2021, the U.S. Food and Drug Administration has been considering NAC as a legal dietary supplement because it has few side effects and potential health benefits. If this proposal is adopted, the long-term intake of NAC as an antioxidant supplement, similar to vitamins, will be possible.

Studies have reported that elevated inflammatory cytokines such as TGF-*β*1, IL-6, MMPs, NO, PGE2, and TNF-*α* stimulate fibrosis, angiogenesis, and intracellular signaling pathways, resulting in LFH progression [4, 9–12, 56, 57]. The p38/MAPK and p65/nuclear factor- (NF-) *κ*B intracellular signaling pathways are also involved in the development of LFH. TGF-*β*1 increases collagen, CTGF, IL-1, and IL-6 expression through the p38/MAPK pathway [17, 53, 58–64]. Furthermore, NF-*κ*B p65 activation engendered an increase in IL-6, IL-1, TGF-*β*, and collagen fiber expression [12, 17, 60, 65]. In addition, myofibroblasts, which are fibroblast phenotypes, constitute a key component of LFH. Myofibroblasts can be differentiated from fibroblasts and protomyofibroblasts on the basis of the stimulation of mechanical stress and TGF-*β*1. Specifically, myofibroblasts generate a stronger contractile force, increase the synthesis of extracellular matrix components such as collagen, and elevate fibrosis-related cytokine expression, which can exacerbate fibrocontractive diseases [66]. Myofibroblast dysregulation results in the progression of fibrosis-related diseases [61]. Studies have reported that in hypertrophic LF cells, especially in the dorsal layer, myofibroblast activity was elevated and that *α*-SMA (a myofibroblast marker) expression was increased and was strongly correlated with increased type 1 collagen expression; moreover, *α*-SMA expression was further increased under hypoxic stimulation [6, 67]. In our study, in hypertrophied LF cells, elevated protein expression levels of p-p38 and p-p65 were detected after a 30 min H_2_O_2_ treatment and the expression levels of iNOS, TGF-*β*, vimentin, and collagen I were elevated after 24 h H_2_O_2_ treatment; this phenomenon is similar to the pathological molecular mechanism of LFH reported in other studies. NAC administration resulted in the regression of inflammatory and fibrotic markers through H_2_O_2_ stimulation. These results suggest that antioxidants could diminish oxidative stress-induced intracellular signaling, inflammation, and fibrosis as well as the potential differentiation of myofibroblasts from fibroblasts.

Changes in cell phenotypes, including the distinct fibroblast phenotypes involved in hypertrophic scar formation, play a role in the development of several diseases [68]. The dysregulation of fibroblasts, myofibroblasts, and macrophages plays a major role in the development of LFH [57]. However, no other studies have considered the diverse phenotypes of LF cells. This study is the first to reveal differences between hypertrophic and healthy LF cells. Specifically, we demonstrated that the hypertrophic LF cells exhibited significantly higher background levels of p38/MAPK signaling and iNOS than did the healthy LF cells. Under oxidative stress, p-p38 and iNOS levels increased more markedly in the hypertrophic LF cells than in the healthy cells, indicating the proinflammatory properties of and highly active intracellular signaling in the hypertrophic cells. This finding suggests that the pathological phenotype of hypertrophic LF cells is different from that of healthy cells. In summary, the pathological status of hypertrophic LF cells is different from that of healthy cells, resulting in an increase in intracellular signaling and inflammatory reactions in such cells under oxidative stress. The increase in baseline endogenous oxidative capacity in hypertrophic LF cells along with the vulnerability of such cells to oxidative stress creates a vicious cycle that may result in the rapid exacerbation of LFH.

Our study has some limitations. First, the age of the LSS and LDH patients was not matched perfectly. Secondly, the study was confined to experiments on cells. Although NAC was verified to have a protective effect against LFH, its low oral bioavailability (4%–10%) and high first-pass effect could engender challenges in its clinical application. Until now, no animal models or clinical trials have applied NAC for LFH treatment. Accordingly, additional clinical studies should be performed to validate the clinical efficacy, therapeutic dosage, and administration route of NAC for LFH. Thirdly, the current study did not cover analyses of posttranscriptional modifications and the secretome associated with LFH. The qPCR of inflammatory markers and ELISA/luminex analyses of inflammatory markers secreted by cultured cells would be performed in the future studies.

## 5. Conclusions

This study revealed that oxidative stress damaged both hypertrophic and healthy LF cells, with the hypertrophic LF cells exhibiting a more active inflammatory response and p38 MAPK pathway than did the healthy cells. This indicates that the pathological phenotype of hypertrophic LF cells has proinflammatory properties. Under oxidative stress, the hypertrophic LF cells exhibited increased expression levels of fibrotic and inflammatory markers and intracellular signaling, and NAC administration reversed the proinflammatory and fibrogenic effects of oxidative stress. These results suggest that antioxidants could form the basis of a novel preventive or therapeutic pharmacologic treatment for LFH. In the future, N-acetylcysteine might be long-term administered as a treatment for LFH based on few side effects and antioxidative property. However, there were some limitations of the study such as the distinct population between LDH and LSS groups and the absence of clinical trial applying NAC for LFH treatment.

## Figures and Tables

**Table 1:** Patient characteristics.

	LDH	LSS	*p* value^∗^
Number	24	28	
Sex (male/female)	17/7	8/20	<0.005
Age (years)	43.1 ± 17.8	67.8 ± 9.2	< 0.001
BMI (kg/m^2^)	26.2 ± 8.3	25.5 ± 4.3	0.707
DM	7/24	4/28	0.19
HTN	6/24	12/28	0.177
Respiratory disease^#^	1/24	1/28	0.911
Smoking	6/24	3/28	0.175
LF thickness (mm)	2.7 ± 0.4	5.1 ± 0.7	<0.001

^∗^ *p* value generated using a chi-square test or independent *t*-test; ^#^respiratory diseases including asthma and bronchiectasis; LDH: lumbar disc herniation; LSS: lumbar spinal stenosis; BMI: body mass index; DM: diabetes mellitus; HTN: hypertension.

## Data Availability

The data presented in this study are available on request from the corresponding author.
